# EXPOSURE TO FINE PARTICULATE MATTER, GLOBAL COGNITIVE PERFORMANCE, AND EMOTIONAL DISTRESS IN OLDER WOMEN

**DOI:** 10.1093/geroni/igz038.3419

**Published:** 2019-11-08

**Authors:** Andrew J Petkus, Xinhui Wang, Diana Younan, Mark M Espeland, JoAnn E Manson, Eric Whitsel, Susan Resnick, Jiu-Chiuan Chen

**Affiliations:** 1 University of Southern California, Los Angeles, California, United States; 2 Wake Forest School of Medicine, Winston-Salem, North Carolina, United States; 3 Brigham & Women's Hospital/Harvard University, Boston, Massachusetts, United States; 4 University of North Carolina, Chapel Hill, North Carolina, United States; 5 National Institute on Aging, Baltimore, Maryland, United States

## Abstract

Among older adults, exposure to ambient PM2.5 (particulate matter with aerodynamic diameter <2.5 μm) has been associated with more rapid decline in cognitive performance and greater emotional distress. However, the inter-relationship between PM2.5 exposure, emotional distress, and global cognitive decline is unexamined. We examined whether long-term PM2.5 exposure affects global cognitive ability and emotional distress in 5,982 older women (baseline age 70.6 ± 3.8 years) from the Women’s Health Initiative Memory Study. PM2.5 exposure for the three-years prior to baseline was estimated at each participant’s residence via a national kriging model. Using structural equation models (SEM), a z-score standardized latent factor consisting of items from the 6-item CESD and the SF-36 Emotional Well-Being scale was constructed to estimate emotional distress at baseline and one-year follow-up. Individual-specific trajectories of global cognitive performance (Modified-Mini Mental State Examination; 3MS) were also estimated. All effects reported were adjusted for multiple demographic, lifestyle, and clinical variables. Higher PM2.5 exposure was associated with lower baseline 3MS performance (β= -.159 per IQR=3.38 μg/m3; 95% CI = -.276 to -.042), which was associated with increased emotional distress over the subsequent year (β= -.011; 95% CI= -.017 to -.004). A statistically significant indirect association of PM2.5 on changes in emotional distress via worse baseline 3MS performance (β=.002; 95% CI = .001 to .004) was present. In contrast, no statistically significant association between PM2.5 on baseline emotional distress occurring prior to declines in 3MS performance was present. PM2.5 neurotoxicity may contribute to global cognitive decline, which precedes increased emotional distress.

